# In Love With the Virus: Reducing Harms, Promoting Dignity, and Preventing Hepatitis C Through Graphic Narratives

**DOI:** 10.1177/15248399211041075

**Published:** 2021-10-19

**Authors:** Aleksandra Bartoszko

**Affiliations:** 1 VID Specialized University, Oslo, Norway

**Keywords:** critical narrative intervention, hepatitis C, comics, injection drug use, substance use, pleasure, ethnography, harm reduction, stigma, equity

## Abstract

This article describes a process of creating an ethnographic comic about injection drug use and hepatitis C, based on long-term ethnographic fieldwork in Norway. The project and the graphic publication titled The Virus were a collaboration between a social anthropologist, a graphic artist, and individuals who inject illegal drugs and are aimed at reducing bodily, social, and narrative harms related to drug use. The article argues that structurally informed interventions, such as this project, which account for the social, economic, and epistemological inequalities, benefit from taking phenomenological perspectives seriously. In our case, that attitude meant including participants’ positive associations with their current or former heroin and injecting drug usage, their stigmatized desires, and their emotions—such as love—related to the disease. The article describes the narrative, conceptual, aesthetic, and practical choices encountered in making The Virus to confront the dominant, authorized narratives in the field of drug use and hepatitis C. We sought to make choices that ultimately would not contribute to the (re)production of the very object of the prevention—stigma related to hepatitis C—but instead would create a new narrative(s) that forged a sense of purpose, recognition, and humanity.

In Norway, approximately 20,000 people have chronic hepatitis C, and 50 to 100 die or receive liver transplantation every year because of the disease. Approximately half of the persons who inject illegal drugs are infected with hepatitis C virus (HCV); they constitute the majority of those who develop chronic hepatitis C (Dalgard, 2015; Devin et al., 2016). To reduce the number of people living with HCV, one must treat both the disease and focus on transmission routes to avoid new infections and reinfections (e.g., Meijerink et al., 2017; Midgard, 2017).

People who inject drugs lack knowledge about infection and virus transmission (Bartoszko & Magnussen, 2016). In fact, HCV infection is rarely mentioned as a risk connected to injecting drugs, even though HIV infection is widely communicated as such a risk, and that both are often co-occurring (Bartoszko & Ponomarew, 2016). Myths about safe injections abound, such as the idea that HCV dies if the injection solution is heated or that the virus can be rinsed off with water or dishwashing detergent. People who use drugs and know the risk of HCV tend to normalize it, attaching little weight to the possibility of infection, partly because many have several more acute challenges.

Recently, the focus in Norway on harm reduction related to injecting drugs and HCV has increased, including the creation of a national hepatitis C strategy and free treatment for all persons infected with HCV, including persons who use drugs (Helse-og omsorgsdepartementet, 2018). However, the measures are not sufficient in terms of volume and type of measures or access to safe injection equipment (Bartoszko & Magnussen, 2016). This deficit is often linked to limited knowledge among those responsible for treatment and preventive work regarding injection practices and the priorities of those who inject drugs. As a result, treatment campaigns and prevention measures are detached from the lifeworlds of affected communities. Successful interventions depend on effective communication with the priority population. Here, effective communication includes respect for often discounted ways of living, which by extension forges another narrative about the reality of people injecting drugs than the one that dominates public health discourse (see, e.g., Malins, 2009).

The graphic ethnography, *The Virus* (Bartoszko & Ponomarew, 2016), was a collaboration between a social anthropologist, a graphic artist, and individuals who inject illegal drugs, currently or formerly. The aim was to reduce HCV infections and encourage testing and treatment. Initially, we sought to create a platform for mutual understanding between recipients and providers of health and harm reduction services. However, as we centered on the practices, logics, and language of affected individuals and took their lived experiences and narratives seriously, we discovered meanings beyond the sheer educational goals of the comic. As we worked on *The Virus*, we forged a mode of ethical engagement, action, and activism that went beyond simple information provision and rational decision making. As a critical narrative intervention, the project became an opportunity to destabilize current ways of understanding these individuals and communicating associated risks.

The aim of this article is twofold. First, I describe the process of creating the ethnographic comic. Here, I do not make a distinction between the critical narrative *processes* and the finished *product*, which I argue cannot be separated. This understanding depends on a broader approach to narrative. Based on the works of Bruner (2003, 2004), Mattingly (2010), and Garro (2009), I emphasize that narratives are not just stories, but rather, they are an “active and constructive mode of cognitive engagement that reflects participation in specific social and moral worlds and depends upon personal and cultural resources” (Garro, 2009, p. 79). According to Garro and Mattingly (2000), narratives “offer a powerful way to shape conduct because they have something to say about what gives life meaning, what is inspiring in our lives, what is dangerous and worth takings risks for” (p. 11). In other words, they are models of and for actions, practices, and behaviors. From that perspective, narratives are not only told stories, but they also are *acted* stories (Mattingly, 2010). Thus, the process of making *The Virus*—not solely the final product—can be seen as part of this acted story, a critical narrative event that changed the persons involved, and in turn, facilitated a new structure for further social actions.

Second, I argue that this critical narrative intervention, which accounts for the social, economic, and epistemological inequalities, benefits from taking phenomenological perspectives seriously. In creating *The Virus*, this meant including participants’ positive associations with their current or former heroin and injecting drug usage, their stigmatized desires, and their emotions—such as love—related to the disease. If we were to ignore the relationship between individuals, their desires, and the stories they want to live, we risked reproducing the stigmatizing discourse of health campaigns and education curricula (Brewis & Wutich, 2019; Lupton, 2015) that are not simply ineffective but harmful for bodies and social relations. Thus, a crucial element of *The Virus* as a critical narrative intervention to shift shaming conversations was to narrate the experiences of people who lived with HCV with aesthetics and language of their own (e.g., Figures 1 and 2).

**Figure 1:**
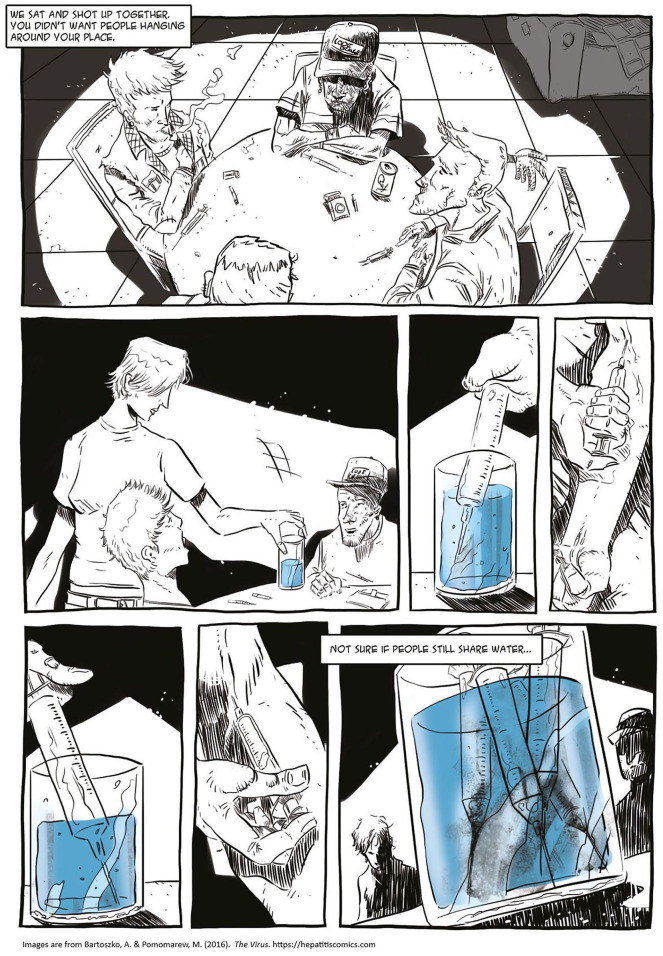
Depictions of the Language and Aesthetics of People Using Injection Drugs

**Figure 2:**
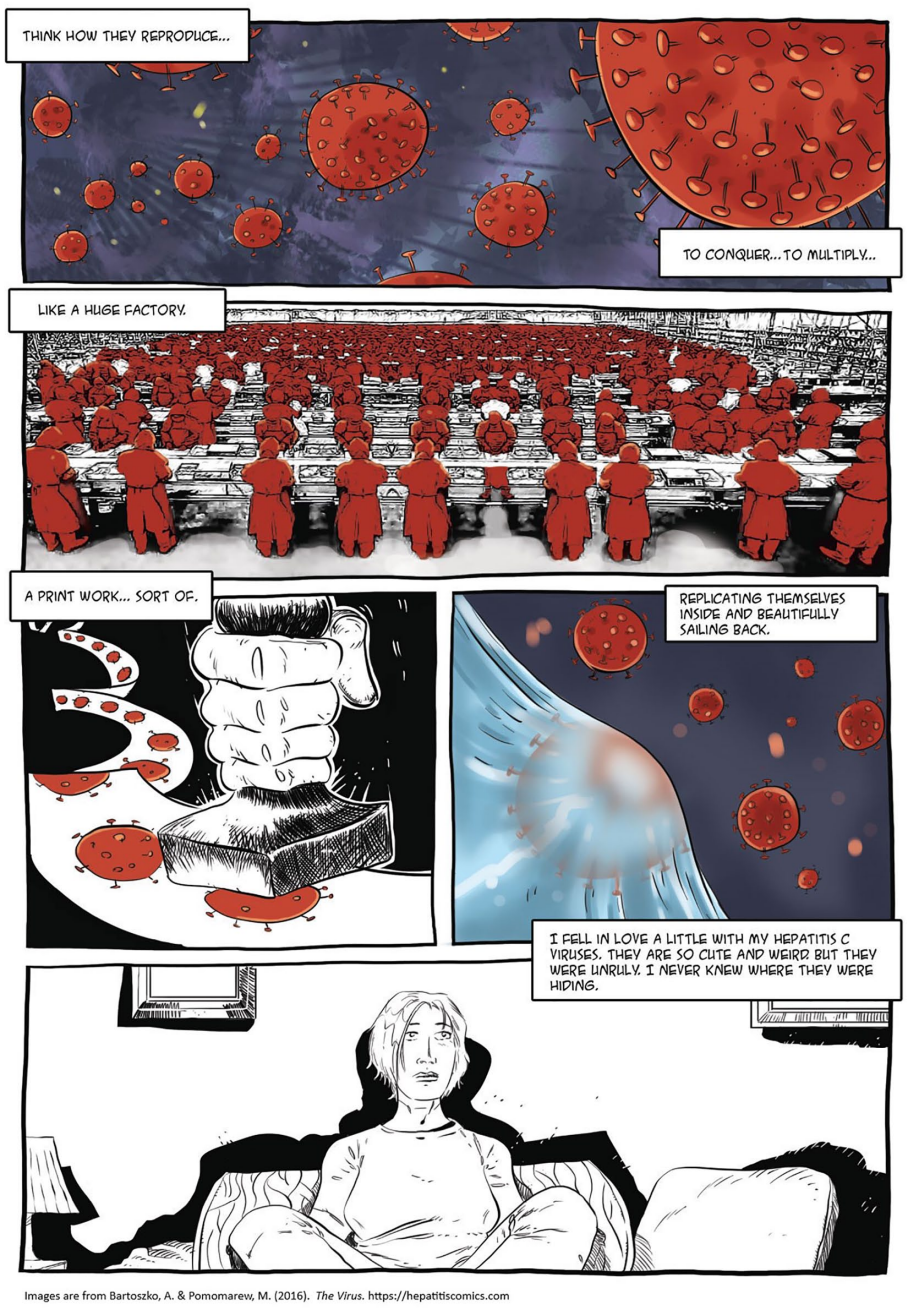
Depictions of the Language, Aesthetics, and Emotions of People Who Live With HCV

This article also investigates the tensions between Harm Reduction as “movement for social justice built on a belief in, and respect for, the rights of people who use drugs,” and harm reduction as a public practice involving “practical strategies and ideas aimed at reducing negative consequences associated with drug use” (National Harm Reduction Coalition, 2021). For instance, the Harm Reduction movement has helped shift stigmatizing narratives about people who use drugs and have been involved with pleasure activism (brown, 2019). *The Virus* identifies with Harm Reduction and as discussed later in the article, challenges harm reduction as a public health strategy that often disregards pleasure and discounts the lived experiences of “targeted populations.” Therefore, this intervention was not an effort to “balance” the participants’ positive associations of drug use with the preventive/harm reduction perspective. Balancing implies that these positive associations are at odds with the public health perspective. On the contrary, we attempted to avoid—in fact, destabilize—this polarized discourse. Increasingly, research has emphasized (Bartoszko, 2021; Duff, 2008; Frank, 2017; Moore, 2008) what my fieldwork also has documented: Drug use is a positive social, cultural, and personal phenomenon that has potential for harm. Attending to the immediate experiences of my interlocutors as they narrated, lived, and cocreated the plot of *The Virus* was central to my learning about the cultural frameworks that serve as resources and hindrances to public health interventions of equality, inclusion, and dignity. Through the ethnographic process, I grew to understand the benefits of including these otherwise discounted knowledges and perspectives in public health discourse.

Next, I describe the narrative, conceptual, aesthetic, and practical choices we encountered in making *The Virus.* We sought to make choices that ultimately would not contribute to the (re)production of the very object of the prevention—stigma related to hepatitis C—but instead would create a new narrative(s) that forged a sense of purpose, recognition, and humanity. As Iren Magnussen,^1^ whose story is told in *The Virus*, expressed in her speech at the comic’s launch, this project “created a space where we can appear as human beings,” and, thus, by extension, recalibrate damaging and disempowering conversations on social health and well-being (Gubrium et al., 2019, p. 291).

## Meeting Iren and The Viruses: Methodological Approach

*The Virus* stemmed from my doctoral work on pharmaceutical treatment of opioid addiction, for which I conducted ethnographic fieldwork among patients and health providers in opioid substitution treatment in Norway. Most of my interlocutors involved with opioid use were infected with HCV, although they rarely appeared to be particularly concerned. As one of them said, “Everybody has it, who cares?” Hepatitis was not a subject in our conversations partly because our relationship focused mostly on their struggles with substitution medication. Additionally, it can take many years for HCV infection to produce disease symptoms and raise concern, as the comic shows.

When I interviewed Iren about her methadone treatment, she was more interested in sharing her HCV experience than others. She enthusiastically talked about the viruses in her body and fantasized on what happened to them while she was undergoing treatment (e.g., Bartoszko & Ponomarew, 2016). Admittedly, this kind of narration of disease was surprising and outside my main focus at that time. However, as an ethnographer, I follow my interlocutors’ logics, fascinations, and lived narratives. Also, this kind of research contains an ethical obligation. Iren’s personal disease and treatment story and her social and political engagement and dream to place HCV on the agenda had an impact on me as a researcher. I was compelled to *act*.

Later, I learned about Iren’s initial skepticism toward researchers, a skepticism she shared with many of my interlocutors who are used to being objectified. At the launch party for *The Viru*s, she related how our relationship and the project began at the needle exchange site and low-threshold clinic in Oslo in September 2016. Here is an excerpt from notes she made for her speech:

2. meeting with an interesting researcher

I shared my experiences [with HCV] with FHN [Norwegian Association for Human Drug Policies, a user organization], where I then sat on the board. We discussed various ideas for knowledge development to disseminate them to active users. Lots of great ideas. Unfortunately, none of them realized.

Until I . . . Abracadabra!! met ALEKSANDRA, here at Switch garden party in the summer of 2014. She introduced herself as a PhD student. At that time, I was naturally a bit skeptical toward those who go around interviewing informant objects to get some filling for their theory understanding (The skepticism was rooted in experiences I had had with some former “master’s degree students”). The skepticism calmed down. Especially after A posted a picture from the Switch garden party and commented: “They do not have such a garden party at Aschehoug.”^2^ It piqued my curiosity. I thought she was an interesting research object. I wanted to talk to her.

First, we talked a bit about OST [opioid substitution treatment], methadone, Dolcontin [morphine], and such. Then it was just hepatitis, hepatitis, hepatitis. To my great delight.

Aleksandra came up with the idea for the comic. She showed me things she had done before. I thought it was a great idea. Almost too good to be true. The FHN board liked the idea and decided that they would support the project.

**Figure 3:**
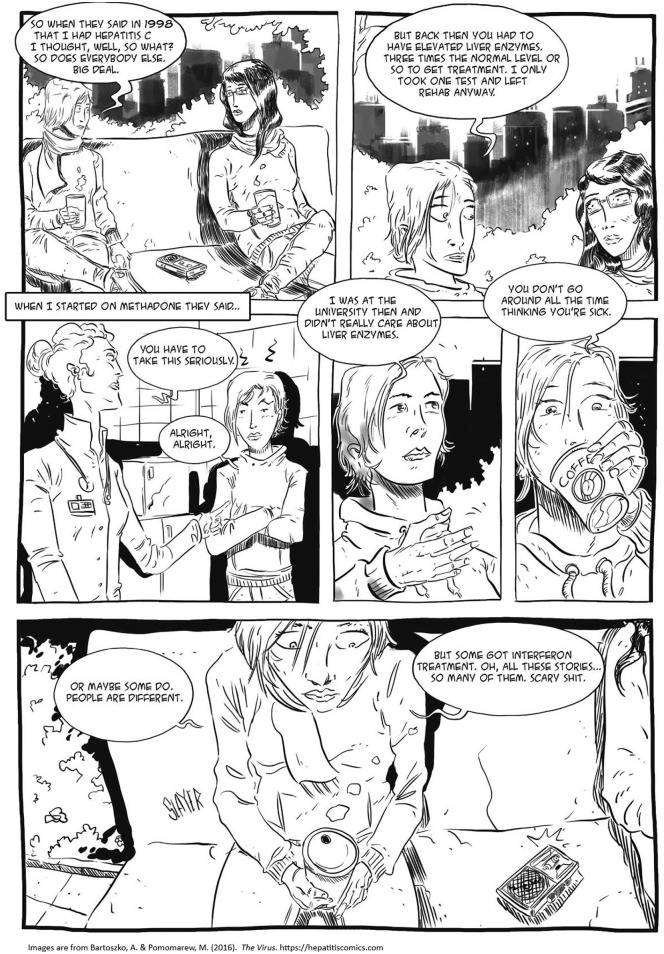
Depictions of the Ethnographic Interview Process

In other words, developing the project required not only my interest in the subject and the people involved but also Iren’s interest in me as a collaborative partner, an individual to explore, understand, and potentially trust. This perspective is important because the current tendency to “involve” affected communities in research projects or interventions implies involving “them” in “our” projects. Such paradigms and contributions often reproduce the hierarchies they are meant to address, and in turn, undercommunicate the researcher’s dependency on communities and the power of the priority population—in this case, Iren’s power to decide and design our relationship and to create the project with me. Therefore, to promote and maintain the awareness of the *narrative codependency* between “interview objects,” we included early in the comic images of us talking and doing interviews (Figure 3). Here, I want to make a caveat. As Iren mentions, it was me who launched the idea of a graphic representation of her experiences during one of our conversations. However, I would have not come up with it on my own, without the dialogues, our relationship, and the reciprocal efforts made after one of us come with an idea during the already ongoing cooperation.

Another issue, which Iren mentioned in her speech, was the project’s founding. I could devote research time to the project through my position at the university; however, the production required financial support. The Norwegian Association for Human Drug Policies (FHN) had expressed interest, and a pharmaceutical company that began manufacturing and marketing new medicines offered its support. We also explored support from public health authorities and academic presses that publish graphic ethnographies. However, some of these avenues posed problems. First, our approach involved critical investigation of the HCV medicine industry, and therefore, cooperation with a pharmaceutical company seemed too complicated and ethically challenged despite the financial potential. Second, we wanted to make the comic available globally and to reach communities with underdeveloped treatment and infrastructures for harm reduction. We also wanted it to be available for free to service users and providers. With those goals, we found publishing with an academic press to be impossible. At that point, we considered funding from FHN to be most compatible with our goals and the needs of the affected communities.

*The Virus* is based on dozens of ethnographic interviews and unstructured conversations with Iren about her story which I then transformed into a coherent plot. At the launch, Iren said, “While working on this project I became aware that I had no idea how I had become infected. I had many questions and a great need to talk about hepatitis C.” Thus, the project became a tool for Iren to deconstruct and understand her past and present (e.g., Bartoszko & Ponomarew, 2016), and according to the narrative approach, shape her future.

As our relationship generated a “project,” we found we needed more data. We worked together to map and reconstruct Iren’s earlier practices. She collected additional data, such as talking to her friends about their current and former injecting practices, while discussing the comic and ideas for how to shape it. I conducted additional in-depth interviews with interlocutors from my doctoral project who had hepatitis C.

After the data collection, I wrote a manuscript/storyboard for the graphic artist Marcin Ponomarew. The text in the comic is quotations from the above-mentioned conversations. We also included a poem written by a person living with HCV and published on an international peer forum for people living with HCV. The artist then made drawing drafts based on the manuscript. Iren and persons from her network read and commented on the storyboard and drawings, guiding revisions regarding the ethnographic details and aesthetic choices. Iren leveraged contacts with a low-threshold hepatitis C clinic, which generated a successful cooperation with specialized health care and other public authorities. As a result, we held a launch at the clinic and distributed the comic in low-threshold facilities, hospitals, and professional associations. We self-published *The Virus* in Norwegian and English. It has been translated, published in other languages, and distributed in countries in cooperation with local harm reduction organizations and public services (e.g., Edekontaminace, 2021).

These processes highlight the power of critical narrative intervention—shifting established and harmful practices *and* stories. This approach required reflexive attention to how particular stories and bodies are portrayed in society, including in other HCV materials and interventions. Which portrayals are adequate and just? In other words, which narratives do we (re)create? We had to determine the linguistic and aesthetic means by which the comic would speak *about* and *to* the lifeworlds of people injecting drugs and the costs associated with such a portrayal and the choices we made. In line with the critical narrative intervention approach, the way we speak about ourselves changes the way we and others think about us. These are ethical choices.

## Against Alienating Interventions

Harm reduction approaches to illicit drug use have proven extremely successful in improving health outcomes for persons using drugs, their families, and the broader community (Irwin & Fry, 2007; Rhodes & Hedrich, 2010). Initiatives such as needle and syringe exchange programs, which provide sterile injecting equipment and health advice, have been established around the world; their success in reducing the spread of blood-borne viruses such as HIV and HCV is well established (Dolan et al., 2005). One reason for their success is that these strategies do not alienate people by judging their habits or pressuring them to stop using drugs.

Nevertheless, many harm reduction programs face strong community opposition, and many others are limited in their effectiveness by the reluctance to acknowledge affective relationships involved in drug use. As Malins (2009) pointed out, “harm reduction, explicitly formulated as a movement of negation (the reduction of harm) rather than one of positive affirmation (the increasing of bodily capacities), necessarily carries with it this tendency to focus on the harmful aspects of drug use” (p. 182). On the other hand, researchers, user organizations, and harm reduction advocates aligned with the Harm Reduction movement promote a more humane approach to drug use and increasingly emphasize the role of pleasure (Bartoszko, 2021; Frank, 2017; Moore, 2008), solidarity (Kavanaugh & Anderson, 2008), bonding (Foster & Spencer, 2013), and ecstasy (Hunt et al., 2010). However, few nationally sponsored health campaigns and harm reduction programs include pleasurable or positive associations with drug use.

Mainstream harm reduction policies and health interventions often overlook that drug use is *valuable* to users. For instance, drug use campaigns and HCV interventions typically refer to the negative associations with drug use and apply tragic narratives and aesthetics that many “targeted individuals” find alienating, stigmatizing, and even oppressing. Here again, I refer to Iren’s memories, which she expressed in her speech during the launch event (excerpt from her notes):

Just before and after starting treatment, I became very interested in acquiring knowledge about hep C. I googled. [. . .] came across x number of pages where hep C was referred to as “the silent killer” and “the world’s 7 deadliest disease.”

It was very interesting but . . . but I felt that something was missing. So that I could better understand/get a grip on the hep C disease . . . That is, what happened in my body.

This unresolved state of knowledge loosened up a bit when I by chance came across [. . .] brochure. And I got to see the picture of the virus. A little beautiful virus star inside me, kind of. Hep C was then transformed (as I say in the comics): “from something diffuse, a name that only exists on a piece of paper to something real, something concrete.”

My experiences during this period thus made me aware of:

1. That knowledge transfer in the form of image and text (in hep C context) suits me better . . . . than JUST text.

2. That I became a little immune to hep C text that triggered my fear receptors (probably the same mechanisms that happen as when one is exposed to such “drug kills” campaigns)

**Figure 4:**
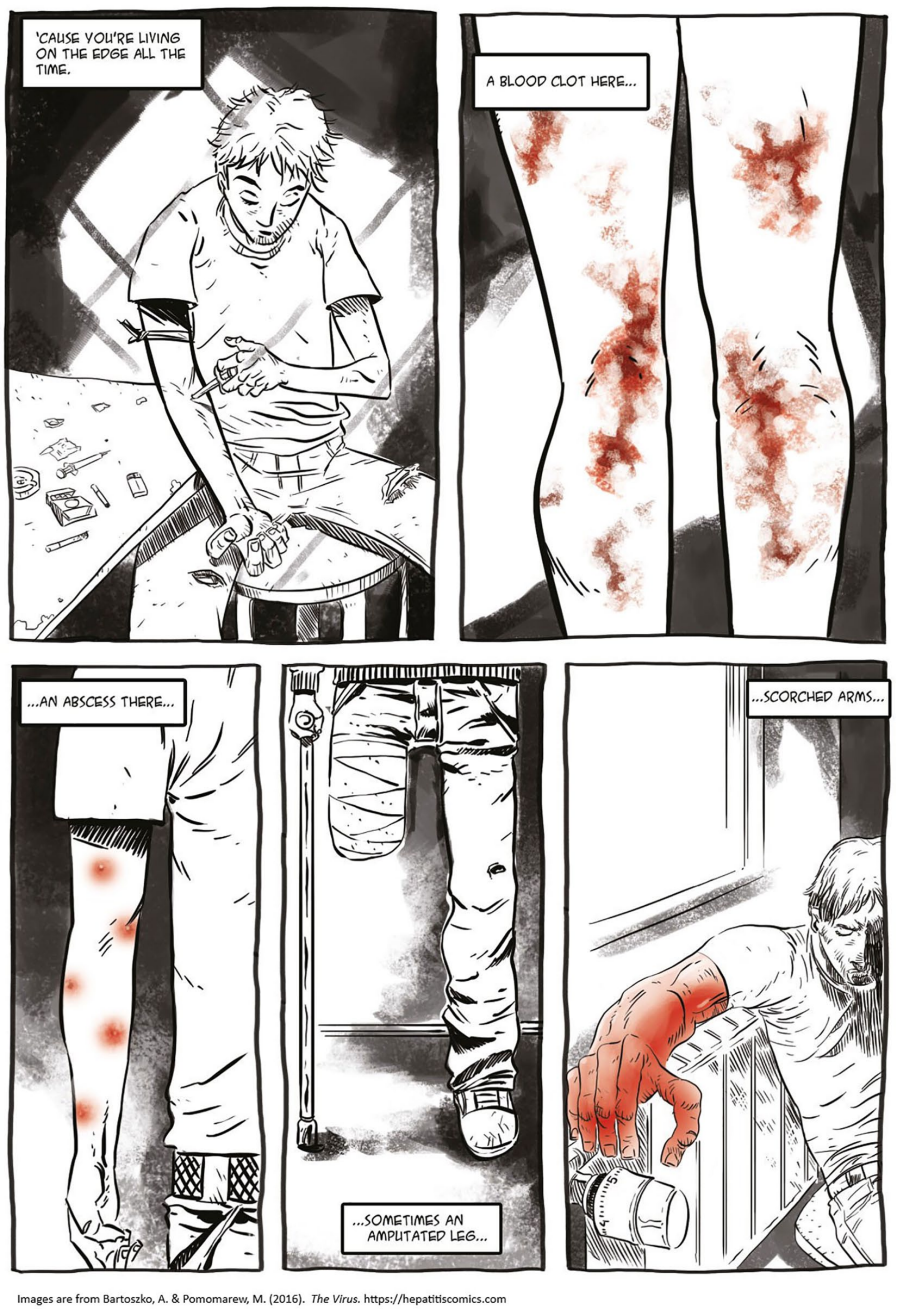
Depictions of Risk Normalization and Emergent Needs of People Who Use Illicit Drugs

Despite her increased focus on HCV, Iren could not find *relevant* and *appealing* information. She was epistemologically and narratively thirsty for “something more.” Her reflections on fear-based health information needed to be considered. In my research before writing the storyboard, I found that Iren and her friends often expressed the desire for an intervention that addressed their needs for narrative and normative recognition. They were often offended by available health materials utilizing infantilizing aesthetics depicting the virus as a kind of monster, vampire, or smiling cartoon figure. Iren and her peers were critical and immune to health information and traditional educative approaches that employ fear of the disease and reveal limited knowledge of risk management, lived experiences, and emergent needs of people who use illicit drugs (Figure 4). Many said they were “fed up with the misery stories,” a dominant narrative, which present use of illicit drugs as a single coherent story leading to “dirt, desperation, and death.”

Indeed, for many persons using illicit drugs, particularly those injecting, misery is *part* of their life story; however, their stories include other elements deserving political and clinical attention. Dominant tragedy narratives and aesthetics on one hand (Tuck, 2009) and success stories of “recovered addicts” on the other hand comprise harmfully reductive narratives about drug use. As Iren and I worked together on this project, we forged a commitment to approach drug use and HCV infection without destructive dramaturgy and excessive communication of risk, and in particular, to avoid cliché portrayals of people who use heroin. To accommodate these considerations, we designed *The Virus* with an intricately layered narrative language that included unauthorized desires and commitments, such as love and an affective relationship toward viruses and heroin use. This was an ethical choice: to refrain from presenting drug use in entirely negative terms and to demonstrate that people who use and enjoy heroin can be reasonable, responsible, *and* affective at the same time.

**Figure 5:**
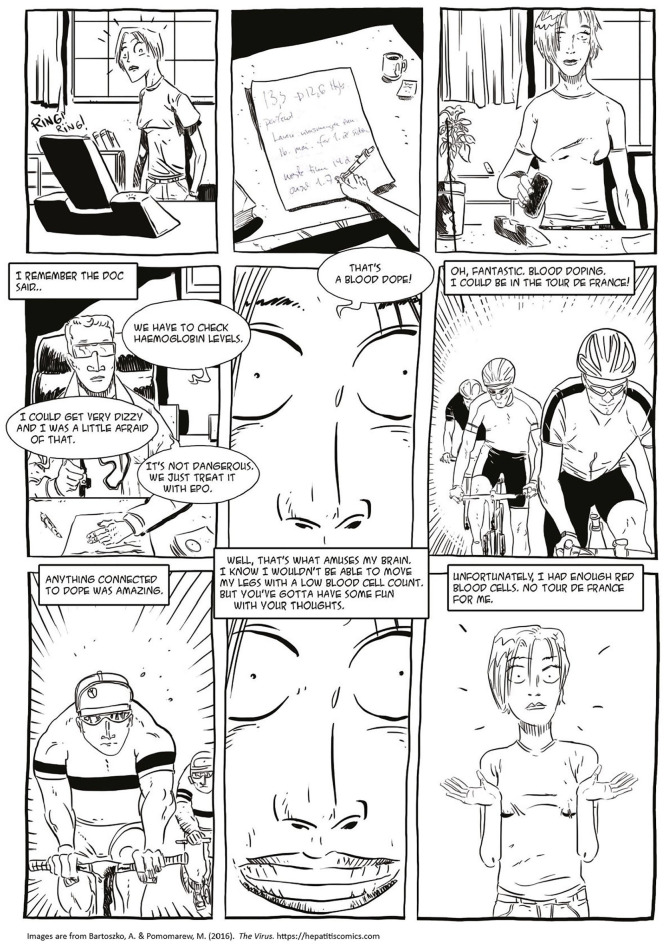
Depictions Normalizing the Image of a Person Using Drugs and/or Living With HCV

Scholars and health educators have emphasized the effectiveness of visual materials, including comics, and have encouraged their use in health and social care (e.g., Green & Myers, 2010; Katz et al., 2006; McNicol, 2017; Park & Zuniga, 2016). However, all images produce social and political effects. Tragic tropes that haunt many drug-referenced advertisements and public health interventions (see Malins, 2009) rely on certain moral preconditions, and reproduce a reductive approach to persons using drugs. By challenging the representational clichés, we wanted to change this unidimensional story of individuals like Iren. Although we indeed included needles, spoons, blood, and dirt in the comic (e.g., Bartoszko & Ponomarew, 2016), these elements are portrayed without sensationalism and excessive narrative and normative layers, and thus, provide a space for both identification and distance. Including humor, such as Iren’s joy over blood medicine used in doping (Figure 5) and applying her own language and metaphors or everyday events such as school reading normalize the image of a person using heroin and/or living with HCV.

Typical drug themes that portray dark, seedy, secret worlds and use shock tactics contribute to “othering” and exoticizing people who use drugs, which further stratifies and segregates them. In her critical analyses of such portrayals in advertising and health and policy materials, Malins (2009) noted the personal, social, and political consequences of this ethico-aesthetics of drug use:

By producing the drug-using subject as diseased, psychotic, unpredictable and irrational, such advertisements work to reduce our capacity and willingness to connect to those who use drugs and to understand their reasons for doing so. As such, they are likely to reduce our ability to comprehend the complexity of the problems surrounding drug use, and our capacity to critically reflect on our own role, via policy and practice, in the emergence and continuation of these problems. This in turn reduces the potential for other connections, such as those of empathy and compassion, and in extension positive intervention. (p. 2)

With these reflections in mind, we developed an aesthetic to accommodate the real practices and lifeworlds of the people without unnecessary demonization of their lifestyles but rather a careful normalization of them.

Drug use involves desire, and we acknowledge it in our intervention. Commenting on the drafts of *The Virus*, a social worker questioned our portrayal of the details in which people may inject drugs. Others were worried that this story might “glorify heroin use,” “encourage drug use,” or “trivialize the critical public health issue of drug addiction.” These are typical reactions that H/harm R/reduction advocates encounter while promoting their work. One argument for showing practices in detail is purely pedagogical and aligns with, for instance, advances in sexuality education, which concludes that we cannot talk about pregnancy without talking about sex. In the same way, we cannot talk about preventing HCV transmission without incorporating injecting practices *as they happen*, no matter if we show them or not.

Another concern we accounted for was contextually relevant preventive interventions, which in many areas have proven to be effective (e.g., Dumka et al., 1998; Leung et al., 2019). In many interventions, the context is understood as social, political, and cultural frames *outside* the individual; however, our understanding of context included individuals’ subjective perceptions, such as their idiosyncratic knowledge and affective relationship toward their own bodies and practices. To take these experiences seriously is to challenge the trivialization of a critical public health issue. The failure to account for other ways of living in public health measures and interventions is a democratic problem because it deprives some individuals and groups from social and political recognition as well as public goods, which *The Virus* attempts to challenge.

If we take phenomenology seriously in critical narrative interventions in health, we include the counterintuitive responses individuals might have, such as, in our case, portraying hepatitis C in terms of love, not trauma or tragedy. Iren developed an affectionate relationship with the viruses inside her. She never characterized the disease as an invader, a popular metaphor for illness. Rather, she said, “I fell in love a little with my hepatitis C viruses. They are so cute and weird, but they were unruly. I never knew where they were hiding” (Figure 2). Her statement contrasts clearly with the dominant narratives in public health literature, which often create a polarized sense of health and illness or well-being, such as “blood is either clean or dirty, pure or defiled, and once hepatitis C has been acquired, cleanliness and purity is the preserve of others only” (Fraser & Treloar, 2006, p. 103). As Fraser and Treloar (2006) pointed out, this narrative tends to frame HCV infection “in terms of an absolute shift from healthy to sick, clean to contaminated, good to bad” (p. 99), which can have significant implications for public health. If someone considers their blood to be completely contaminated, they may worry less about avoiding future bacterial or viral infections, including HCV or HIV (Fraser & Treloar, 2006). Also, this narrative shapes social relations, contributing to “othering” of people with HCV and reflecting shame and an abject self-image.

**Figure 6:**
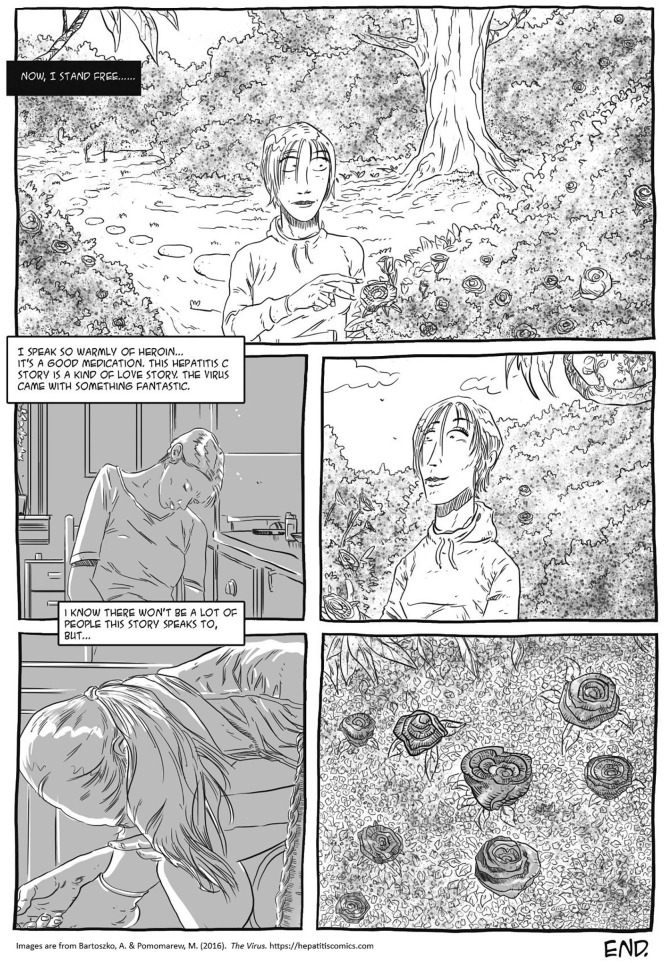
Depictions Reframing Shaming and Tragedy Narratives of Heroin Use and HCV to Narrative of Affection

In addition, Iren explained that she was attracted to the viruses *because* she was infected during her active use of heroin. She loved heroin and praised it for saving her life. Heroin was the best medication, she said (Figure 6), adding that this is not a story that speaks to many people. Again, the way we and others speak about us changes the way we think about ourselves. Individuals who contributed to this project wanted to change the narrative. Therefore, we told this story with the language Iren used not to exoticize but to promote a sense of social connection between the story and persons in a similar situation as Iren. To include Iren’s statements about her love for heroin on the last page of the comic was to explicitly tell what is forbidden *and* to offer Iren’s meta-reflection on how this story breaks with the dominant narrative, creating a “narrative shock” (Krause & Gubrium, 2019) that might change the way Iren and her friends perceived themselves.

As individuals, we enter social situations equipped with culturally shaped ideas of our possible lives in a given context at a given time. We are on a constant quest to create our future and to live out multiple possible futures (Bruner, 2004; Mattingly, 2010). Therefore, people are meaningful agents who are both constituted by a complex matrix of stories and also have any number of stories of their own to tell *and* enact (Mattingly, 2010). *The Virus* needed to be told. By taking desires and affects seriously, we also took individual responsibility seriously, empowering people who use drugs to make reasonable choices while still injecting. We challenged the binary opposition between ideal “rational” subjectivity and the abject irrational “other.” Through this intervention, the individuals involved and those who read the comic may create possible selves as they reshape harm reduction conversations in a more productive direction.

## Implications for Health Promotion Practice

Our approach, we hope, may inspire ethico-aesthetic modes of engagement with drug use, not only among those who consume drugs but also among people around them, such as professionals working with harm reduction and hepatitis C treatment and those in broader society. As Malins (2009) suggested, by using

aesthetic forms of information delivery, educational programs can more affectively engage people on an embodied, non-conscious level. In doing so, however, such aesthetic forms of engagement need to ensure that they engage with a positive ethics based on affirmation, rather than nihilism, and focused on maximising, rather than reducing, bodily capacity. (p. 186)

I define *The Virus* as a Harm Reduction initiative not because it is a tool for information about safe injection, infections, and treatment, but because it addresses structural narrative harms. As Malins (2017) concluded, “Discourses of pleasure do make an important intervention into and against dominant narratives of risk, harm, and addiction” (p. 126). We aimed to increase the representation of not only a historically silenced population but also their stifled perspectives. Through critical narrative intervention, we provoked important questions to guide a new way of harm reduction interventions and showed that acknowledgment of the values, knowledges, and affects of the individuals involved provides a more sensitized, dignity-based approach.

Iren emphasized often that it was not enough to *feel* part of the project but to *be* part of it rather than exterior to it. I will let her conclude this article with the last paragraph of her speech at the launch party:

Thank you for allowing me—in such an elegant way—to combine heavy theory with empathy, commitment, and relationship. You have challenged the role of researcher. You have given a lot of yourself. Which has led to the creation of a space where we can appear as human beings.

## Notes

1. This is not a pseudonym. Iren’s name appears in the comic and she preferred to have her name mentioned on the last page of the comic, where her role is stated. We participated together during public events and we wrote an article together based on this project (Bartoszko & Magnussen, 2016).

2. Iren was referring to the launch of the project “Switch Your Habits,” which aimed to promote smoking heroin instead of injecting it. The launch was held at the yard of the safe injection and needle exchange site in Oslo. Around the same time, a major Norwegian publisher, Aschehoug, organized its annual garden party for the Norwegian cultural elite.
